# CHANGES IN COGNITIVE FUNCTION AMONG SENIOR AMERICANS: TRENDS AND DIFFERENCES BY RACE

**DOI:** 10.1093/geroni/igz038.2413

**Published:** 2019-11-08

**Authors:** Keqing Zhang, Wei Zhang, Yanyan Wu

**Affiliations:** University of Hawai’i at Mānoa, Honolulu, Hawaii, United States

## Abstract

Improvements in health and increase in life expectancy have contributed to the increasing proportion of older population over the past century. It is estimated that by 2050, the number of older adults with cognitive impairments in the United States will increase by 2.5-4 fold, while age-specific rates remain constant. This paper uses data from 10 waves (1996-2014) of the Health and Retirement Study (N= 33213) to crystalize the trends in cognitive function changes and cognitive impairment rates in a nationally representative sample of older adults. OLS and logistic regressions are used to estimate the trends and determine the contribution of sociodemographic variables to decreasing trends in the prevalence of cognitive impairment over time. Results show that with the increase of age, the cognitive function of older adults decline in all races, after adjustment for age, gender, education, and other sociodemographic factors. Also, the annual decline rate of cognitive function is larger for African Americans and Hispanic Americans, while smaller for white and other races. A further investigation of the possibility of cognitive impairment reveals a different scenario: as individual ages, the Hispanic are the least likely to suffer from cognitive impairment, followed by the white, other and black. Improvements in educational level contribute to declines in cognitive impairment across all races, particular the Hispanic Americans. Race-specific findings suggest that future research need to take into account the racial diversity and possibly cultural influences when examining the cognitive functions of older adults.

